# On Disordered Digestion and Dyspepsia

**Published:** 1889-10

**Authors:** 


**On Disordered Digestion and Dyspepsia.** By FRANK WOODBURY, A. M., M. D., Fellow of the College of Physicians of Philadelphia; Honorary Professor of Clinical Medicine in the Medico-Chirurgical College of Philadelphia, etc., etc. 12mo, pp. 82. Detroit, Mich.: George S. Davis. 1889. Price, in, paper, 25c.; in cloth, 50c.

**Syphilis of the Nervous System.** By H. C. WOOD, M. D., LL. D. 12mo pp. 135. Detroit, Mich.: George S. Davis. 1889. Price, in paper, 25c.; in cloth, 50c.

These two brochures are a continuation of the well-known Physician's Leisure Library. The subjects they treat upon are of sufficient importance, and the authors are so reputable, as to commend them at once, without comment in detail, to the studious physician. The enterprise of the publisher, in putting forth such valuable literature in so cheap a form, and with such typographical excellence, is deserving of all praise. They should find ready sale.

